# Causal association between telomere length and female reproductive endocrine diseases: a univariable and multivariable Mendelian randomization analysis

**DOI:** 10.1186/s13048-024-01466-5

**Published:** 2024-07-15

**Authors:** QiaoRui Yang, JinFu Zhang, ZhenLiang Fan

**Affiliations:** 1 Department of Gynecology, Guanghua Hospital Affiliated to Shanghai University of Traditional Chinese Medicine, Shanghai, China; 2 Shanghai University of Traditional Chinese Medicine, Shanghai, China; 3 Department of Gynecology, Shanghai Guanghua Hospital of Integrated Traditional Chinese and Western Medicine, Shanghai, China; 4 Nephrology Department, The First Affiliated Hospital of Zhejiang Chinese Medical University (Zhejiang Provincial Hospital of Chinese Medicine), Zhejiang, China; 5 Academy of Chinese Medical Science, Zhejiang Chinese Medical University, Zhejiang, China

**Keywords:** Mendelian randomization, Leukocyte telomere length, Female reproductive endocrine diseases, Causality, Genetics

## Abstract

**Background:**

The relationship between leukocyte telomere length (LTL) and female reproductive endocrine diseases has gained significant attention and research interest in recent years. However, there is still limited understanding of the exact impacts of LTL on these diseases. Therefore, the primary objective of this study was to investigate the genetic causal association between LTL and female reproductive endocrine diseases by employing Mendelian randomization (MR) analysis.

**Methods:**

Instruments for assessing genetic variation associated with exposure and outcome were derived from summary data of published genome-wide association studies (GWAS). Inverse-variance weighted (IVW) was utilized as the main analysis method to investigate the causal relationship between LTL and female reproductive endocrine diseases. The exposure data were obtained from the UK Biobanks GWAS dataset, comprising 472,174 participants of European ancestry. The outcome data were acquired from the FinnGen consortium, including abnormal uterine bleeding (menorrhagia and oligomenorrhea), endometriosis (ovarian endometrioma and adenomyosis), infertility, polycystic ovary syndrome (PCOS), premature ovarian insufficiency (POI) and premenstrual syndrome (PMS). Furthermore, to account for potential confounding factors such as smoking, alcohol consumption, insomnia, body mass index (BMI) and a history of pelvic inflammatory disease (PID), multivariable MR (MVMR) analysis was also conducted. Lastly, a series of pleiotropy tests and sensitivity analyses were performed to ensure the reliability and robustness of our findings. *P* < 0.0063 was considered to indicate statistically significant causality following Bonferroni correction.

**Results:**

Our univariable MR analysis demonstrated that longer LTL was causally associated with an increased risk of menorrhagia (IVW: odds ratio [OR]: 1.1803; 95% confidence interval [CI]: 1.0880–1.2804; *P* = 0.0001) and ovarian endometrioma (IVW: OR: 1.2946; 95%CI: 1.0970–1.5278; *P* = 0.0022) at the Bonferroni significance level. However, no significant correlation was observed between LTL and oligomenorrhea (IVW: OR: 1.0124; 95%CI: 0.7350–1.3946; *P* = 0.9398), adenomyosis (IVW: OR: 1.1978; 95%CI: 0.9983–1.4372; *P* = 0.0522), infertility (IVW: OR: 1.0735; 95%CI: 0.9671–1.1915; *P* = 0.1828), PCOS (IVW: OR: 1.0633; 95%CI: 0.7919–1.4278; *P* = 0.6829), POI (IVW: OR: 0.8971; 95%CI: 0.5644–1.4257; *P* = 0.6459) or PMS (IVW: OR: 0.7749; 95%CI: 0.4137–1.4513; *P* = 0.4256). Reverse MR analysis indicated that female reproductive endocrine diseases have no causal effect on LTL. MVMR analysis suggested that the causal effect of LTL on menorrhagia and ovarian endometrioma remained significant after accounting for smoking, alcohol consumption, insomnia, BMI and a history of PID. Pleiotropic and sensitivity analyses also showed robustness of our results.

**Conclusion:**

The results of our bidirectional two-sample MR analysis revealed that genetically predicted longer LTL significantly increased the risk of menorrhagia and ovarian endometrioma, which is consistent with the findings from MVMR studies. However, we did not notice any significant effects of LTL on oligomenorrhea, adenomyosis, infertility, PCOS, POI or PMS. Additionally, reproductive endocrine disorders were found to have no impact on LTL. To enhance our understanding of the effect and underlying mechanism of LTL on female reproductive endocrine diseases, further large-scale studies are warranted in the future.

**Supplementary Information:**

The online version contains supplementary material available at 10.1186/s13048-024-01466-5.

## Introduction

Telomeres, which are nucleoprotein structures located at the ends of linear chromosomes in eukaryotic cells, are composed of highly repetitive DNA sequences and shelterin proteins and belong to heterochromatin regions [1]. The main role of telomeres is to safeguard the integrity of chromosomes by preventing fusion and degradation, playing a crucial role in DNA repair and maintenance of genomic stability [2, 3]. However, during mitosis, incomplete replication of the lagging strand leads to the loss of DNA at the 3' end of the chromosome. This phenomenon, known as the "end-replication problem", leads to the gradual shortening of telomeres with each cell division [4]. Eventually, the telomere shortening triggers cell proliferation arrest and senescence [5], which is particularly common in most somatic cells where telomerase activity is almost lost [4]. Nonetheless, in germ cells, stem cells, embryonic cells and 90% of tumor cells, telomere shortening is prevented due to the presence of telomerase activity, ensuring a broader capacity for cell proliferation [4, 6]. Telomere length (TL) is a complex hereditary trait that is initially established in germ cells and passed down to the fertilized egg [7]. Leukocyte telomere length (LTL) serves as an easily measurable indicator in peripheral blood and is generally correlated with TL in most tissues, making it a proxy for TL in other parts of the body [7]. During early life, LTL wears down rapidly, but slows down to an annual shortening rate of approximately 25–35 base pairs after reaching adulthood [8]. Notably, short LTL has been proven to be associated with various pathological conditions, including coronary heart disease, type 2 diabetes mellitus, Alzheimer's disease, among others [9–11]. On the other hand, longer LTL is primarily linked to multiple types of cancers [12, 13].

Reproductive endocrine diseases encompass a range of common gynecological conditions, such as abnormal uterine bleeding (menorrhagia and oligomenorrhea), endometriosis (ovarian endometriomas and adenomyosis), infertility, polycystic ovary syndrome (PCOS), premature ovarian insufficiency (POI), premenstrual syndrome (PMS), etc. In recent years, numerous studies have highlighted a strong association between TL and these reproductive endocrine disorders. Xu et al. [14] and Miranda-Furtado et al. [15] observed shorter LTL in peripheral blood of POI women compared to the control group, accompanied by shortened TL and relatively decreased telomerase activity in granular cells. Conversely, Sayban et al. [16] noticed significantly longer TL in whole blood samples of POI individuals. Velazquez et al. [17] found that after adjusting for age, LTL was significantly longer in PCOS women compared to the controls. However, a recent meta-analysis indicated no difference in LTL between PCOS and non-PCOS populations [18]. Dracxler et al. [19] discovered that the TL of lymphocytes in patients with endometriosis was significantly longer, possibly 8.1 times longer than in normal individuals. In contrast, a retrospective study conducted by Sasamoto et al. [20] found a correlation between shorter LTL and an increased risk of endometriosis. Although these studies reveal the link between TL and female reproductive endocrine diseases, unfortunately, the directionality of these evidence is not consistent. Therefore, conducting large-scale research to explore the correlation between TL and various reproductive endocrine diseases holds significant clinical significance and potential applications.

Mendelian randomization (MR) is a robust method used in the field of genetics to examine the causal effects of modifiable exposures on health outcomes. By utilizing genetic variations, usually single-nucleotide polymorphisms (SNPs), as instrumental variables (IVs), MR is able to minimize the influence of confounding factors and reverse causality bias [21]. This is achieved based on the application of Mendel's second law (law of segregation), where DNA is passed from parents to offspring during gamete formation, resulting in independent segregation of alleles and random distribution of genetic variations. It is similar to the random allocation in randomized controlled trials, which is designed to create groups with comparable clinical characteristics, thus minimizing the potential confounding factors and providing more accurate analytical results [22, 23]. Since the genetic variants are unmodifiable and unaffected by disease status, MR studies have also gained attention in reducing the risk of reverse-causation bias [24, 25]. Furthermore, MR analyses allow for the investigation of exposures that have an expected adverse effect on disease risk, which may be unethical to study in the clinical trials [24, 25]. All in all, MR analysis offers a means to address some of the limitations and obstacles encountered in traditional observational studies and randomized controlled trials. Multivariable MR (MVMR), as an emerging extension of MR, incorporates multiple genetic risk factors that may be associated with the exposure-outcome relationship into the model, thereby reducing the potential biases caused by confounding factors to certain extent [26]. Based on this, employing the MR method to explore the risk factors for female reproductive endocrine diseases can develop more effective prevention and intervention strategies.

In conclusion, in order to overcome the limitations of small sample sizes as well as the challenges in study design and implementation typically associated with observational research, this study utilized extensive genome-wide association study (GWAS) data and employed a bidirectional two-sample MR combined with MVMR analysis method to unveil the causal relationship between LTL and female reproductive endocrine diseases, which not only offers substantial and compelling evidence but also presents novel avenues for clinical decision-making.

## Materials and methods

### Study design

**Fig. 1:**
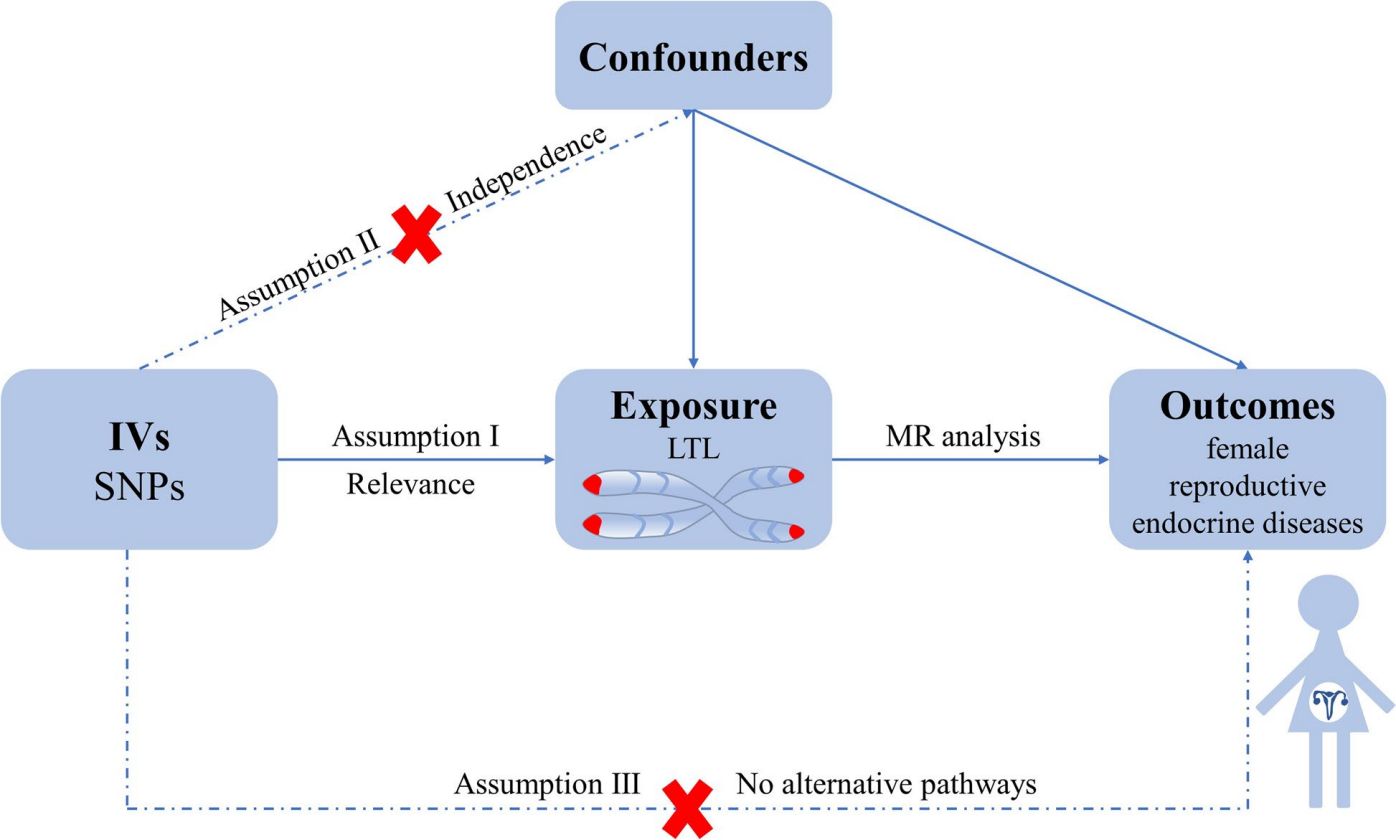
Assumptions of MR analysis for LTL and the risk of female reproductive endocrine diseases. IVs: instrumental variables; SNPs: single-nucleotide polymorphisms; LTL: leukocyte telomere length; MR: Mendelian randomization

We implemented a two-sample MR analysis with data obtained from different large-scale GWAS datasets to investigate the casual relationship between LTL and eight female reproductive endocrine diseases. The methodology employed in our study was illustrated in Fig. 1. To estimate causal effects, the selection of potential genetic variants in our study must satisfy three key hypotheses [24]: (1) Assumption I (relevance assumption): The genetic instrumental variables are strongly correlated with the exposure of interest (LTL), thereby minimizing bias associated with weak instrumental variables. (2) Assumption II (independence assumption): The genetic instrumental variables are independent of any known or unknown confounders that might mediate the relationship between exposure (LTL) and outcome (female reproductive endocrine diseases). This independence can be assessed by determining whether the genetic variant is linked to competing risk factors. (3) Assumption III (exclusion restriction assumption): Genetic variants influence the outcome solely through the exposure, with no remaining direct causal pathways. Since our study utilized data extracted and screened from published and accessible GWAS summary statistics, which had already been approved by ethical review boards, ethical approval was not required.

### Data sources

The genetic instrumental variables used in this study for the exposure factors were derived from the largest previously published GWAS. These GWAS provided information on LTL and 20,134,421 SNPs from 472,174 participants of European ancestry in the UK Biobanks [27]. LTL refers to the average length of leukocyte telomeres which was determined through a multiplex quantitative polymerase chain reaction assay on a diverse population of leukocytes [27]. GWAS summary data for menorrhagia (23,458 cases and 111,583 controls), oligomenorrhoea (1,150 cases and 111,583 controls), adenomyosis (4,665 cases and 111,583 controls), ovarian endometrioma (6,444 cases and 111,583 controls), infertility (14,759 cases and 111,583 controls), PCOS (3,676 cases and 223,193 controls), POI (542 cases and 218,970 controls) and PMS (295 cases and 111,583 controls) were acquired from the FinnGen consortium R10 version (https://r10.finngen.fi/) [28].

In addition, we obtained pooled data on other factors such as current tobacco smoking (GWAS ID: ukb-b-223), alcohol consumption (GWAS ID: ukb-a-25), BMI (GWAS ID: ukb-a-248), sleeplessness/insomnia (GWAS ID: ukb-a-13) and inflammatory diseases of female pelvic organs (GWAS ID: finn-b-N14_FEMALEGENINF). These data were sourced from Neale Lab or MRC-IEU consortium, etc., ensuring that all GWAS sample populations analyzed in our study are of European ancestry. The details of all relevant datasets are provided in Table S1.

### Genetic instrumental variables selection and evaluation

In order to meet the three hypotheses mentioned earlier, the following steps were taken to screen the IVs. Initially, SNPs that displayed a significant association with LTL were selected, specifically those with a *P*-value below 5 × 10^−8^. Subsequently, we eliminated SNPs that exhibited linkage disequilibrium, meaning SNPs with an r^2^ greater than 0.001 with the most significant SNPs within a cluster window of 10,000 kb range. Furthermore, we performed a sequential screening through PhenoScanner V2 (http://www.phenoscanner.medschl.cam.ac.uk/) to identify potential confounders with horizontal pleiotropy at the genome-wide significance level [29]. Finally, to mitigate the risk of weak instrument bias, R^2^ and F values were individually calculated for each SNP to evaluate the reliability of the IVs. F was computed using the formula F = R^2^ × (N − 2)/(1 − R^2^), where R^2^ represents the proportion of variability in exposure explained by each IV and N denotes the total sample size of GWAS for sources of SNPs strongly associated with exposure [30, 31]. R^2^ was calculated by the formula R^2^ = 2 × EAF × (1 − EAF) × β^2^/[(2 × EAF × (1 − EAF) × β^2^) + (2 × EAF × (1 − EAF) × N × SE^2^), where EAF refers to the frequency of the effect allele, βrefers to the estimated genetic effect on the exposure, N refers to the total sample size of GWAS where a strong association between SNPs and the exposure was found, and SE refers to the standard errors of genetic effects [30, 32]. SNPs with an F-statistic greater than 10 were considered to have a low likelihood of weak instrument bias and were included in the final analysis.

Given the evidence from previous studies highlighting the adverse effects of unhealthy lifestyles, such as smoking, drinking and staying up late, as well as high BMI and a history of pelvic inflammatory disease (PID) on the female reproductive endocrine system, we conducted MVMR analysis to adjust for genetic liability to the aforementioned risk factors, using the same IV screening procedures and criteria as mentioned above.

### Statistical analysis

The inverse-variance weighted (IVW) was considered as the primary statistical approach to assess the causal relationship between exposure (LTL) and outcome (female reproductive endocrine diseases), and other analytical methods included mendelian randomization-egger regression (MR-Egger), Weighted median, Weighted mode and Simple mode. The IVW method generally assumes that genetic variants are valid and uncorrelated with each other. Its principle is mainly based on the meta-summaries of the causal effects from multiple SNPs with the calculation of Wald ratio [33, 34]. MR-Egger measures mean pleiotropy between IVs by an intercept term and allows for the influence of mechanisms other than the exposure on outcome variables [35]. A non-zero MR-Egger intercept regression with a *P*-value < 0.05 indicates the presence of gene pleiotropy [35]. The Weighted median method relies on a median weighting strategy, requiring at least 50% of the genetic information to be derived from valid IVs, and provides somewhat robust results [36]. Weighted mode and simple mode serve as complementary methods to IVW, providing more reliable estimates in a broader context.

To further illustrate the causal effect of LTL on female reproductive endocrine diseases, we performed MVMR analyses, adjusting for smoking, alcohol consumption, sleeplessness/insomnia, BMI and a history of PID according to previous studies. The MVMR analysis was conducted using both IVW and MR-least absolute shrinkage and selection operator (Lasso) methods. In MR-Lasso, the lasso-type penalization is used to assess the direct impact of genetic variants on the outcome. The resulting causal estimate, known as the post-lasso estimate, is obtained through the IVW method by solely utilizing the genetic variants identified as valid by the lasso procedure, which is an extension of the IVW model [37].

Pleiotropy was assessed primarily through MR-Egger intercept analysis, with a *P*-value < 0.05 indicating horizontal pleiotropy [38]. To examine the heterogeneity of IVs, IVW and MR-Egger were employed, and the results were evaluated using Cochrane's Q value, with a *P*-value < 0.05 hinting the existence of heterogeneity. Sensitivity analysis was performed using mendelian randomization pleiotropy residual sum and outlier (MR-PRESSO) after removing outliers to further ensure the stability and reliability of our MR results [38, 39]. The funnel plot was also drawn as a visually display of the sensitivity analysis results, helping to identify any abnormal points based on the symmetry. Finally, we conducted a leave-one-out analysis by systematically removing SNPs to further determine their specific impact on the MR results.

All statistical analyses were performed using R (version 4.3.1, https://www.r-project.org/), with a nominal causality significance threshold of *P* < 0.05 (two-sided). After the Bonferroni correction test, *P* < 0.0063 was considered to indicate robust evidence of causality with significant statistical significance, in order to account for multiple testing. MR analysis with IVW, MR-Egger, Weighted median, Weighted mode, Simple mode and MR-PRESSO was conducted by R packages “Two Sample MR (version 0.5.8)” and “Mendelian Randomization (version 0.9.0)”. MVMR was conducted by R packages “Mendelian Randomization (version 0.9.0)” and “MVMR (version 0.4)”. The statistical power of MR was calculated using the online tool mRnd: Power calculations for Mendelian Randomization (https://cnsgenomics.shinyapps.io/mRnd/), with a recommended power greater than 80%.

## Results

### Genetic instrumental variables

In the present study, a total of 140 SNPs were preliminarily identified as IVs for LTL, which served as the exposure variable in the MR analysis. The range of the F-statistic for these genetic instruments varied from 29.860 to 1105.847, indicating that all the IVs had sufficient validity. The R^2^ for the 136 SNPs explained 0.006324% to 0.233657% variance range. Additionally, further screening using PhenoScanner did not reveal any potential confounders or results associated with these SNPs. After excluding four SNPs (rs2276182, rs2306646, rs56178008 and rs670180) for being palindromic with intermediate allele frequencies or missing in the outcome, there were 136 SNPs included in the MR analysis (Table S2). The statistical power of menorrhagia, oligomenorrhoea, ovarian endometrioma and POI exceeded 80%, suggesting strong reliability in the results. However, the statistical power of adenomyosis, infertility, PCOS and PMS was lower, showing a higher chance of false negatives (Table S3).

When investigating different female reproductive endocrine diseases as exposures in the MR analysis, we initially identified 70, 13, 31, 73, 27, 16, 9 and 3 SNPs as IVs for menorrhagia, oligomenorrhoea, adenomyosis, ovarian endometrioma, infertility, PCOS, POI and PMS, respectively. After removing SNPs with missing outcomes and palindromic sequences, we retained 64, 12, 29, 64, 22, 12, 8, and 3 SNPs for the MR analysis of menorrhagia, oligomenorrhoea, adenomyosis, ovarian endometrioma, infertility, PCOS, POI and PMS on LTL, respectively. The F-statistic for all the IVs was greater than 10, suggesting a low possibility of weak instrument bias (Tables S4-S11).

### Univariable MR analysis of the causal relationship between LTL and female reproductive endocrine diseases

**Fig. 2:**
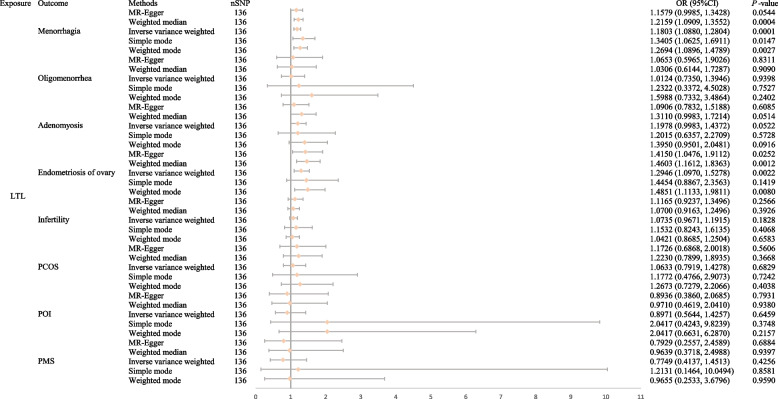
Forest plots of univariable MR results for genetically predicted LTL on the risk of female reproductive endocrine diseases. LTL: leukocyte telomere length; PCOS: polycystic ovary syndrome; POI: premature ovarian insufficiency; PMS: premenstrual syndrome; nSNP: number of single-nucleotide polymorphisms

**Fig. 3:**
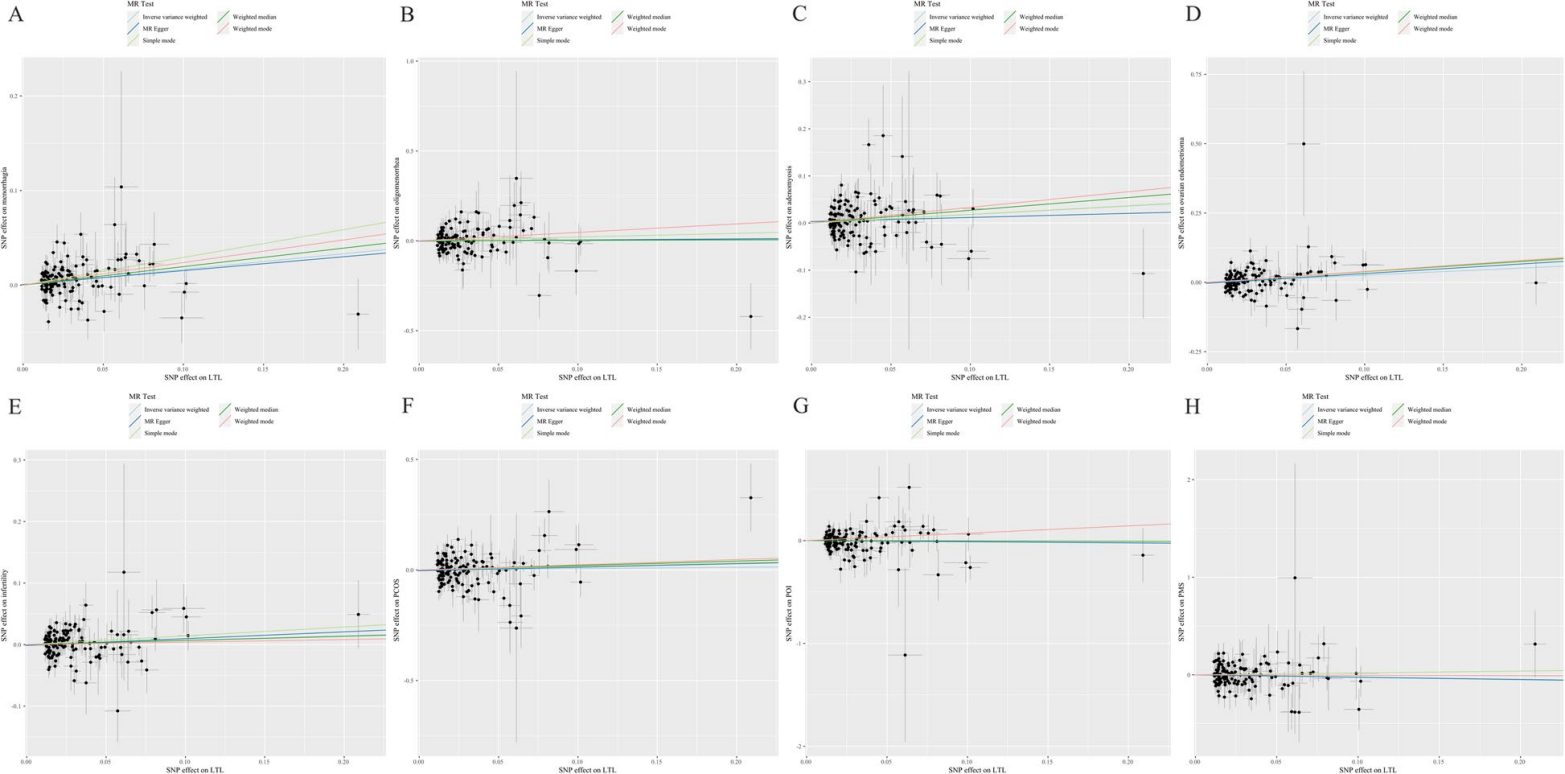
Scatter plots of univariable MR results for the correlation between LTL and female reproductive endocrine diseases. **A** menorrhagia; **B** oligomenorrhea; **C** adenomyosis; **D** ovarian endometrioma; **E** infertility; **F** polycystic ovary syndrome (PCOS); **G** premature ovarian insufficiency (POI); **H** premenstrual syndrome (PMS)

As depicted in Fig. 2, our analysis revealed that longer genetically predicted LTL was associated with an increased risk of two female reproductive disorders in individuals of European ancestry after the Bonferroni correction. Specifically, menorrhagia (OR:1.1803; 95%CI:1.0880–1.2804; *P* = 0.0001) and ovarian endometrioma (OR:1.2946; 95%CI:1.0970–1.5278; *P* = 0.0022) showed a positive correlation with LTL using the IVW method. Additionally, the Weighted median (OR: 1.2159; 95%CI: 1.0909–1.3552; *P* = 0.0004), Simple mode (OR: 1.3405; 95%CI: 1.0625–1.6911; *P* = 0.0147), and Weighted mode (OR: 1.2694; 95%CI: 1.0896–1.4789; *P* = 0.0027) methods supported the findings of the IVW method for menorrhagia. In the case of ovarian endometrioma, the MR-Egger (OR:1.4150; 95%CI:1.0476–1.9112; *P* = 0.0252), Weighted median (OR:1.4603; 95%CI:1.1612–1.8363; *P* = 0.0012), Simple mode (OR:1.4454; 95%CI:0.8867–2.3563; *P* = 0.1419) and Weighted mode (OR:1.4851; 95%CI:1.1133–1.9811; *P* = 0.0080) methods provided consistent results with the IVW method. However, no statistically significant associations were observed between LTL and oligomenorrhoea, adenomyosis, infertility, PCOS, POI or PMS using any of the five statistical methods. The scatter plots of the relationship between LTL and eight female reproductive endocrine diseases calculated with five methods are exhibited in Fig. 3, which show consistent directionality.

Furthermore, when investigating the potential causal impact of the eight female reproductive endocrine diseases on LTL with MR analysis, no evidence of a causal relationship was found (Figure S1). The results obtained from the IVW, MR-Egger, Weighted median, Simple mode and Weighted mode methods remained consistent (*P* > 0.05).

### Sensitivity analysis

Sensitivity analysis was primarily evaluated based on the results of IVW, MR-Egger, MR-Egger intercept analysis and MR-PRESSO, as shown in Table S12, to detect potential heterogeneity and horizontal pleiotropy. In all cases, the MR-Egger intercept analysis did not yield statistically significant results (*P* > 0.05), indicating an absent presence of directional pleiotropy. However, the Cochrane's Q test, MR-Egger test and MR-PRESSO all suggested varying degrees of heterogeneity, consistent with the findings in the funnel plots (Figure S2). To assess the influence of individual SNPs on the overall causality assessment, leave-one-out analysis was conducted, wherein each SNP was removed separately and the MR analysis was repeated. Notably, no single SNP was found to have a significant impact on the causality results in the leave-one-out plots (Figures S3). Despite the presence of some heterogeneity in our study, the fact that the MR-Egger intercept was not significantly different from 0 and no SNP with a significant effect on causal estimation was identified in the leave-one-out plots, indicates that the heterogeneity does not significantly affect the reliability of our findings. Therefore, our results remain robust.

### Multivariable MR analysis of the causal relationship between LTL and female reproductive endocrine diseases

In the MVMR analysis, we conducted adjustments for several pertinent variables, including current tobacco smoking, alcohol consumption, BMI, insomnia and PID (Table S13). Our research indicated that the causal relationship between LTL and menorrhagia or ovarian endometrioma remained significant in both univariate and multivariate adjusted models. Furthermore, the results of the MR-Lasso test also supported these findings. In addition, the MR-Lasso test results revealed a significant statistical association between LTL and PCOS, but only after adjusting for current tobacco smoking, alcohol consumption and BMI separately. The MVMR-Egger and IVW sensitivity analyses showed varying degrees of heterogeneity, but none had a significant impact on horizontal pleiotropy, thus indicating the robustness of the results (Table S14).

## Discussion

By performing a large-scale MR analysis of LTL and female reproductive endocrine diseases utilizing data from IEU OpenGWAS project database and FinnGen database, we investigated the effect of LTL on various reproductive endocrine diseases, including abnormal uterine bleeding (menorrhagia and oligomenorrhea), endometriosis (ovarian endometrioma and adenomyosis), infertility, PCOS, POI and PMS. Our findings indicated that longer LTL is associated with an increased risk of menorrhagia and ovarian endometrioma. However, the significant associations between LTL and oligomenorrhea, adenomyosis, infertility, PCOS, POI and PMS were not observed. Ovarian endometrioma, defined as the presence of ectopic endometrial glands and stroma in unilateral or bilateral ovaries, is a type of generalized endometriosis and an estrogen-dependent disease that is characterized by chronic pelvic pain, dyspareunia and infertility [40]. Aligning with our findings, Hapangama et al. [41] and Dracxler et al. [19] have also noticed the correlation between longer TL in peripheral blood and endometriosis. On the contrary, retrospective studies using data from the National Health and Nutrition Examination Survey and New England Case–Control Study database have suggested a link between shorter LTL and a higher susceptibility to endometriosis [20, 42], and the telomeres have shortened by 1% with every passing year since the diagnosis of endometriosis [42]. Notably, the inconsistent findings may be attributed to the reliance on subjective patient self-reports and the lack of reliable pathological reports or other diagnostic evidence for the definition of endometriosis within the database.

In addition, numerous studies have reported that the length of telomeres in the endometrium changes with the ovarian hormone cycle. During the progesterone-dominated secretory phase, the endometrial TL is significantly shorter compared to the proliferative phase, accompanied by a decrease in telomerase activity (TA) [43–45]. Conversely, estrogen can induce the enhancement of TA in endometrial tissues, effectively delaying the shortening of TL [45]. After ovulation, the concentrations of estrogen and progesterone in the peritoneal cavity are 5 to 10 times higher than those in the blood [46]. Besides, the ectopic endometrial tissues exhibit resistance to progesterone [47], suggesting that the high local concentrations of estrogen and progesterone antagonism in the abdominal cavity may activate TA and lengthen TL in ectopic endometrial tissues. Studies by Valentijn et al. [43] further supported our hypothesis that in patients with endometriosis, the relative average TL in ectopic glandular-like epithelium is longer, and TA is at a high level, which means that ectopic endometrial glands have a greater proliferative capacity and a longer survival period. These factors may contribute to the highly aggressive and recurrent nature of endometriosis. Hapangama et al. [48] observed that ectopic endometrial tissues in the peritoneum undergo continuous replication without DNA breaks, which is different from the characteristics of endometrial tissues in healthy women. This phenomenon may be related to the protective effect of the telomere-telomerase system. However, some studies have reported that TL in peripheral blood is shorter than that in endometrium, hinting possible tissue-specific regulation of TL [43]. Additionally, patients with endometriosis exhibit elevated levels of inflammatory factors in peripheral blood [49, 50]. Chronic systemic inflammation is known to increase leukocyte turnover, thereby accelerating telomere attrition rate [51]. This may be a significant factor contributing to the observed shorter TL in peripheral blood compared to those in endometrial tissues. Further observational studies are still needed to clarify whether there are differences in TL properties between the peripheral and reproductive systems.

TA plays a crucial role in maintaining TL and preserving chromosomal homeostasis. The catalytic subunit telomerase reverse transcriptase (hTERT) is one of the three core subunits of human telomerase, with the other two being dyskerin protein and the telomerase RNA component (hTERC) [52]. Previous studies have revealed that compared to healthy females, women with endometriosis display higher levels of TA, hTERT and hTERC during the secretory phase of the endometrium. These characteristics are associated with longer average TL in the endometrium and may contribute to aberrant proliferation of endometrial tissues during this phase, independent of dyskerin [41, 44, 48, 53, 54]. This suggests a disturbance in the homeostasis of periodic proliferation and secretory changes in the endometrial tissues, leading to sustained proliferative potential. Additionally, Kim et al. found that the levels of hTERT mRNA and TA increase with the severity of endometriosis [54], indicating that the telomere-telomerase system not only contributes to the pathological features of endometriosis but also reflects the disease severity. Thus, it has the potential to serve as a biomarker for endometriosis and early identification of these changes could lead to more precise and targeted treatment strategies, thereby improving patient outcomes. Hapangama et al. [55] detected positive immunoreactivity for telomerase in ectopic endometriotic tissues of humans and in both ectopic and eutopic endometriotic tissues of baboons. Notably, the immunoreactivity of proliferative markers, including telomerase, in baboon eutopic endometrial tissues was activated after the intrapelvic injection of menstrual endometrium and sustained for up to 15 months, indicating the development of a proliferative phenotype contributing to the establishment of endometriosis [55]. While telomerase inhibitors can effectively inhibit the growth, migration and invasion characteristics of ectopic lesions from endometriosis [56]. Therefore, we propose that both eutopic and ectopic endometrial tissues from patients with endometriosis exhibit proliferation beyond replicative senescence. This mechanism is related to increased TA and its protective effect on TL, ensuring the dynamic characteristics of endometrial cells.

Interestingly, adenomyosis, formerly known as endometriosis interna, refers to the presence of ectopic endometrial tissues in the myometrium, which shares histological similarities with endometriosis and is also considered as an estrogen-dependent disease [57]. However, no association between LTL and adenomyosis was observed in our study, which is consistent with the observations of Gleason et al. [42]. Maoga et al. [58] discovered membrane type-2 and type-3 matrix metalloproteinases changes in endometriotic entities, but not in adenomyotic or eutopic endometrial tissues with or without endometriosis, suggesting that the alternations in the invasion of ectopic endometrial implants occur after implantation, rather than before. This finding is consistent with the study by Löffelmann et al. [59] and may be attributed to the differences between the intrauterine and intra-abdominal environments. Additionally, retrograde menstruation occurs in the majority of women, but only about 10% and 19.5% of women suffer from endometriosis and adenomyosis [60, 61], which raises the question of whether the implantation of ectopic endometrium is determined by the environment in which it is ectopic, rather than the ectopic endometrium itself.

On the other hand, some scholars have proposed that the formation of adenomyosis results from the repeated tissue damage and repair. This hypothesis is based on the observations that activated platelets could drive pathological changes such as fibroblast-to-myofibroblast transdifferentiation, epithelial-mesenchymal transition, extracellular matrix deposition, smooth muscle metaplasia by activating TGF-β/Smad signaling pathway [62], which may lead to the disruption of the endometrial-myometrial interface (EMI) [63]. Furthermore, no significant difference in E-cadherin and N-cadherin expression was observed in the eutopic endometrium of adenomyosis subjects compared to healthy women, but N-cadherin expression was higher in ectopic lesions than in healthy endometrium [64]. At the same time, adenomyosis lesions showed lower levels of apoptosis and autophagy compared to eutopic endometrium [65]. These findings suggest that abnormal cell survival and invasion at the lesions may be involved in the occurrence of adenomyosis after EMI disruption. Besides, the immunostaining response of progesterone receptors was significantly attenuated in endometrial stroma and ectopic lesions from patients with adenomyosis [65]. Given that the inhibition of TA is a downstream effect of progesterone signaling [43], this hints that local TA and TL may be abnormal in these lesions. Thus, the telomere-telomerase system may be involved in the pathological processes following EMI disruption. However, this pathological process may not be adequately explained by LTL alone, and this may also account for the lack of observed relationship between LTL and adenomyosis in this study.

Based on these findings, we propose that the activation of telomere-telomerase system occurs more generally in ectopic endometrial tissues in the abdominal cavity, and that LTL serves as a reliable indicator of this alteration. In contrast, the occurrence of adenomyosis is more likely related to the disruption of the endometrial-myometrial junctional zone barrier, the invagination of the endometrial basalis, the metaplasia of displaced embryonic pluripotent Mullerian remnants, or the differentiation of adult stem cells [57, 66]. LTL does not accurately represent the local pathological changes of adenomyosis. However, more experimental studies are still needed to further explore the different histopathological characteristics of ectopic endometrium in endometriosis and adenomyosis, as well as the role of telomere-telomerase system activation, in order to provide new insights for clinical treatment. Overall, these findings provide evidence that endometriosis and adenomyosis are not different phenotypes of the same disease, at least from the perspective of the telomere system.

The association between LTL and PCOS has been a topic of controversy in previous studies. However, the present study aligns with the findings of Wei et al. [67] as it found no relationship between LTL and PCOS. In contrast, Wang et al. [68] observed longer LTL in the peripheral blood of PCOS patients, which positively correlated with serum testosterone levels. Although some studies have reported that overweight and obesity could potentially contribute to the shortening of LTL in the peripheral blood of women with PCOS, it is important to note that the LTL in PCOS still remains longer than that in healthy women with similar BMI [69]. In addition, in young and normal-weight women with PCOS, there is evidence of a negative correlation between TA in peripheral blood and BMI [70], indicating that BMI may affect LTL by inhibiting TA. On the other hand, Li et al. [71] discovered that PCOS patients had shorter LTL compared to the control group that was 6.16 years older, and LTL was negatively correlated with serum dehydroepiandrosterone sulfate levels. Surprisingly, Pölönen et al. [72] did not observe any leukocyte telomere attrition in PCOS patients aged 31 and 46 years. Similarly, Pedroso et al. [73] reported that in cumulus cells of mature and immature oocytes, TL was not significantly different between PCOS and healthy populations, but TA was significantly increased in PCOS individuals. Conversely, Yu et al. [74] noted shorter TL in cumulus cells, accompanied by decreased levels of luteinizing hormone receptor and androgen receptor, showing that the positive effects of androgen on telomere maintenance may be repressed. Moreover, TL was significantly prolonged in granulosa cells (GCs) of PCOS patients [67]. Oppositely, Li et al. observed shorter TL and no significant difference in TA in GCs of PCOS [75]. Unfortunately, these studies did not simultaneously detect and analyze the levels of androgen and insulin in PCOS populations, because previous literature reported the negative effect of insulin on TA in GCs of PCOS patients and the positive effect of androgen on TA [17, 76]. In addition, some studies have found that metformin can prolong LTL in PCOS patients, although the difference was not statistically significant, possibly due to the small sample size [77].

These inconsistent conclusions may be attributed to the variations in diagnostic criteria for PCOS, differences in age, metabolic status, medication use and the timing and methods of TL testing. Additionally, the impact of PCOS complications on TL and TA has also often been overlooked. Future large-scale population-based studies are necessary to provide further clarity on the regulatory and pathological characteristics of the telomere-telomerase system in different populations and cell types. Therefore, given the complex pathological changes and diverse clinical subtypes of PCOS, it appears that the association between LTL and PCOS cannot be evaluated in a straightforward manner.

Similarly, the correlation between LTL and POI remains inconclusive. It has been proposed that longer TL in peripheral blood is linked to a reduced number of cell divisions in the early germ cell pool and that premature depletion of follicular resources contributes to POI [78]. Furthermore, since POI patients often require oral estrogen therapy, this may effectively protect telomeres from direct oxidative damage while promoting TA, thereby repairing the telomeres and delaying telomere attrition [16, 78]. Despite this, numerous studies still support the association between telomere shortening and POI as well as ovarian aging [79, 80], accompanied by a decrease in TA [81]. Reduced TA may imply a diminished capacity to repair telomeres, resulting in increased telomere attrition and limited cell proliferation, which is crucial for the development and maturation of follicles. In fact, no association between LTL and POI was found in our research. LTL reflects the replication history of cells and the systemic aging of the body [82]. However, ovarian aging occurs more rapidly than that of other organs [83]. Although the LTL in peripheral blood is easy to obtain and measure, it may not accurately represent TL in other cell types that are more directly related to POI [84]. This discrepancy may explain why the genetically predicted association between LTL and POI was not observed in our study. Further studies on TL and TA involving larger populations are needed to elucidate the connection between LTL and reproductive system aging.

Although our study did not report a direct link between LTL and infertility, previous research has highlighted the significant role of telomeres in various aspects, including follicular development, fertilization and embryo implantation [85]. Hapangama et al. found that there was no significant difference in the average TL of the endometrium during the implantation window across different types of reproductive failure populations, but there was specific expression of TA [86]. Moreover, it was observed that the average TL in peripheral blood lymphocytes of infertile patients was significantly shorter than that of healthy donors of the same age, exhibiting more extreme telomere loss and doublets [87]. Short telomeres could also constrain germ cell specification while promoting differentiation towards somatic lineages [88]. Given the inherent disparities in telomere biology between somatic cells and germ cells, particularly within the microenvironment of follicular growth and development and peripheral blood cells, it is essential for further research to be conducted in order to comprehend these biological mechanisms. This would ultimately facilitate the development of more comprehensive laboratory-to-bedside assessments for both ovarian aging and infertility.

Furthermore, while a relationship between LTL and menorrhagia was observed, this association was not noted for oligomenorrhea and PMS. Gene variants related to LTL may specifically participate in the pathophysiological processes underlying menorrhagia, which is often linked to abnormal endometrial proliferation, myometrial anomalies and abnormal uterine artery blood flow, among other related factors > [89, 90]. These processes may involve systemic factors such as inflammation, oxidative stress and cellular aging, which are reflected in LTL [72, 91] PMS is generally thought to be influenced by fluctuations in GABA conductance and neurosteroid levels [92], which are often more susceptible to external environment and present transient characteristics, inconsistent with the long-term systemic processes predicted by the telomere biology. And beyond that, considering the close interplay between sex hormones and the aforementioned disorders, as well as their impact on TL and TA, further experiments are required in the future to illustrate the biological mechanisms connecting these factors. These findings will enhance our understanding of female reproductive endocrine disorders and provide valuable insights for their prevention and treatment.

Human aging is characterized by a gradual loss of long telomeres and an accumulation of short telomeres, with younger populations exhibiting significantly longer TL compared to older individuals [93]. Similar to this biological relationship, age also plays a crucial role in female reproductive endocrine disorders. Advancing age is frequently associated with a decline in both the quantity and quality of follicles [94]. It is worth noting that the symptoms of female reproductive endocrine related diseases are typically alleviated or disappear after menopause period. In this study, the median age of first onset for various female reproductive endocrine diseases ranged from 29 to 44 years (Table S15), indicating a relatively young age. However, the absence of age data for the control subjects poses a potential confounder. If the control subjects are generally older than case subjects, the natural age-related decline in LTL may obscure the relationship between the disease and LTL. Younger case subjects may possess longer telomeres, which could be mistakenly attributed to the disease process rather than age-related factors. Therefore, caution should be exercised when interpreting the causal relationship between LTL and female reproductive endocrine diseases. Future research should aim to investigate the association between LTL and female reproductive endocrine diseases across different age groups to yield more definitive conclusions.

This study highlights several significant strengths that enhance its contribution to existing research on the relationship between LTL and various female reproductive endocrine diseases. Firstly, this study investigated the causal relationship between LTL and a range of conditions, including menorrhagia, oligomenorrhoea, adenomyosis, ovarian endometrioma, infertility, PCOS, POI and PMS at the genetic level. By adopting a bidirectional MR analysis, this study effectively avoids the measurement biases and confounding factors often inherent in observational studies. Secondly, we utilized extensive GWAS datasets and selected genetic variants for IVs based on the most comprehensive GWAS data available, which ensures sufficient statistical power and excludes the influence of weak IVs, thereby enhancing the robustness of the results. Thirdly, a series of sensitivity analyses and pleiotropy tests were conducted to further support our findings, which are crucial in minimizing potential biases, thereby enhancing the reliability and significance of our conclusions. However, there are some limitations to this study. Firstly, MR analyses can only be applied to risk factors for which appropriate genetic variants are available, and genetic variants typically exert only a small influence on most risk factors, which can result in low statistical power and an increased risk of false-negative results. Moreover, using genes as IVs inevitably introduces biases such as developmental compensation, and the limited availability of extensive GWAS summary data as well as the protection of individual privacy restrict the ability to perform stratified analyses based on variables such as age, BMI, menopausal status and menstrual cycle, which may also cause biases. Thirdly, it is worth noting that the GWAS data utilized for LTL analysis comprised a proportion of men (45.8%) and women (54.2%), potentially introducing bias into our results. Lastly, all the summary data used in our analysis were derived solely from European populations, thereby limiting the generalizability of our findings to individuals from different ethnic backgrounds. Future studies should aim to validate these results in diverse ethnic groups.

## Conclusion

In conclusion, our MR analyses provide robust evidence of a genetic causal role of LTL in female reproductive endocrine disorders. ​Specifically, our findings suggest that longer LTL is associated with a higher risk of menorrhagia and ovarian endometrioma, even after conducting MVMR analyses. This innovative research enhances our understanding of LTL's involvement in the pathogenesis of reproductive endocrine diseases and indicates that LTL could potentially serve as a biomarker for menorrhagia and ovarian endometrioma. However, further research is still necessary to investigate and validate the exact role and underlying biological mechanism of LTL in the occurrence and development of reproductive endocrine diseases in diverse populations, so as to provide a basis for clinical prevention strategies and treatments.

### Supplementary Information

Supplementary Material 1.

Supplementary Material 2.

## Data Availability

No datasets were generated or analysed during the current study.
