# Supplementary material for: Causal association between telomere length and female reproductive endocrine diseases: a univariable and multivariable Mendelian randomization analysis
Source: J Ovarian Res. 2024 Jul 15;17:146. doi: 10.1186/s13048-024-01466-5 (PMC11247788; doi:10.1186/s13048-024-01466-5)
Supplement: Supplementary file 1 — Supplementary Material 1. [file 13048_2024_1466_MOESM1_ESM.docx]

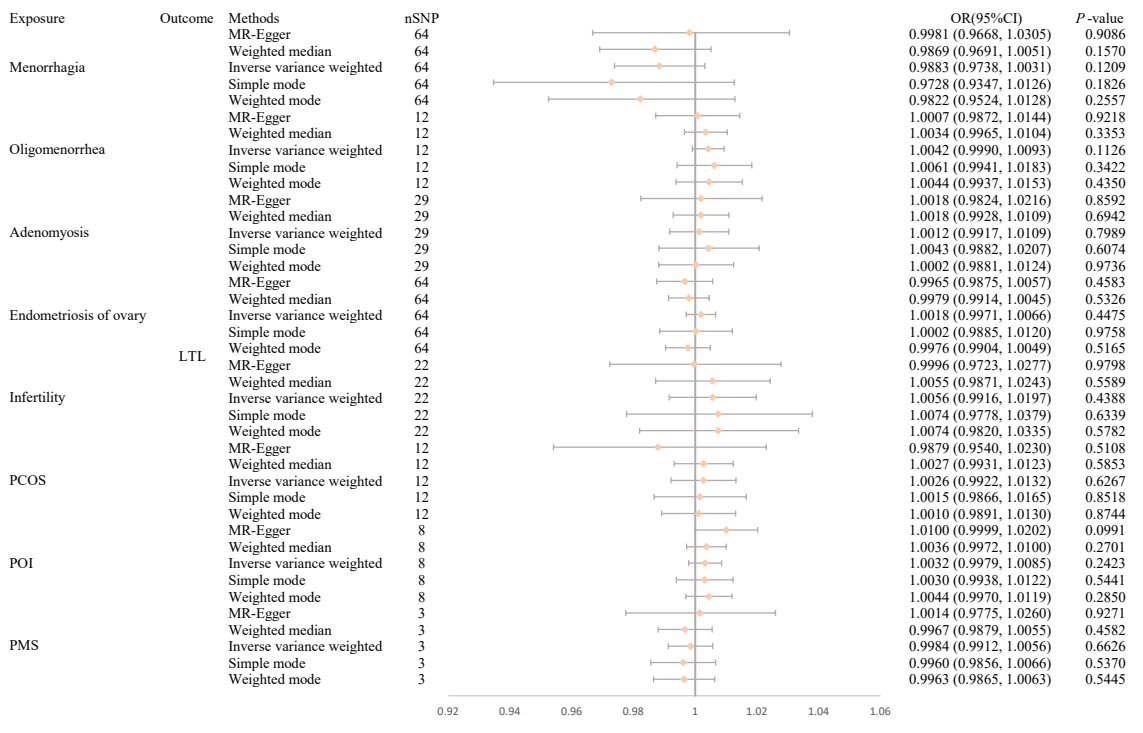


Figure S1. Forest plots of reverse MR results for the relationship between female reproductive endocrine diseases and LTL. LTL: leukocyte telomere length; PCOS: polycystic ovary syndrome; POI: premature ovarian insufficiency; PMS: premenstrual syndrome; nSNP: number of single-nucleotide polymorphisms.


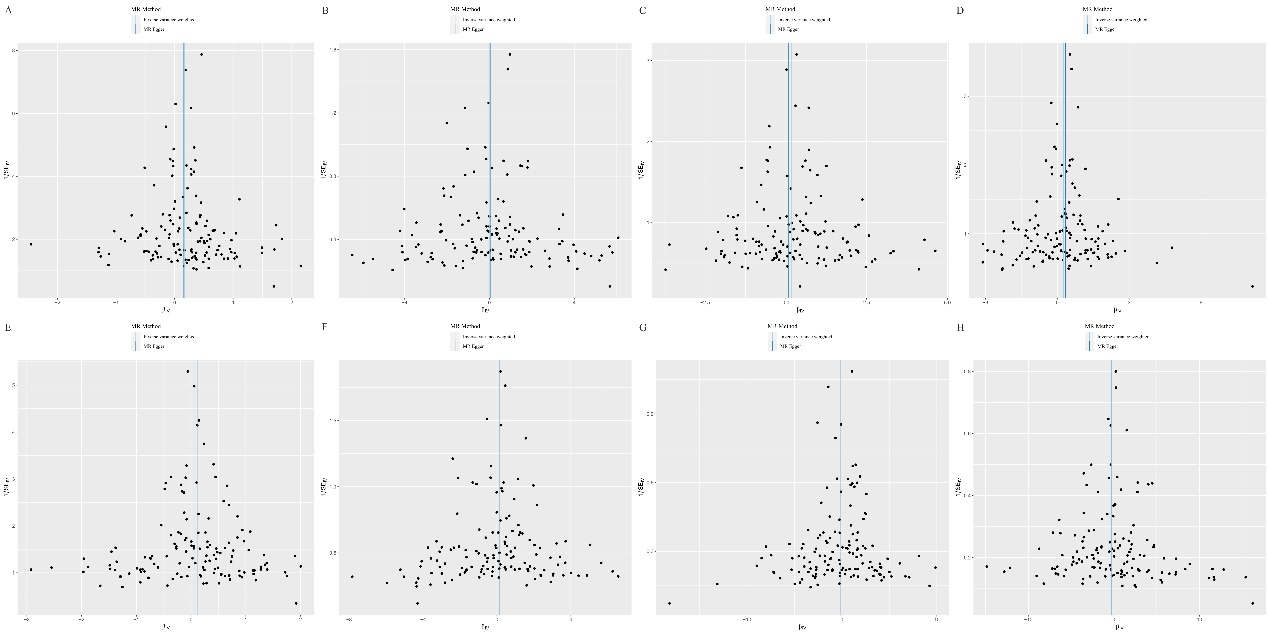


Figure S2. Funnel plots of MR results for the relationship between female reproductive endocrine diseases and LTL. (A)menorrhagia; (B)oligomenorrhea; (C)adenomyosis; (D)ovarian endometrioma; (E)infertility; (F)polycystic ovary syndrome (PCOS); (G)premature ovarian insufficiency (POI); (H)premenstrual syndrome (PMS).


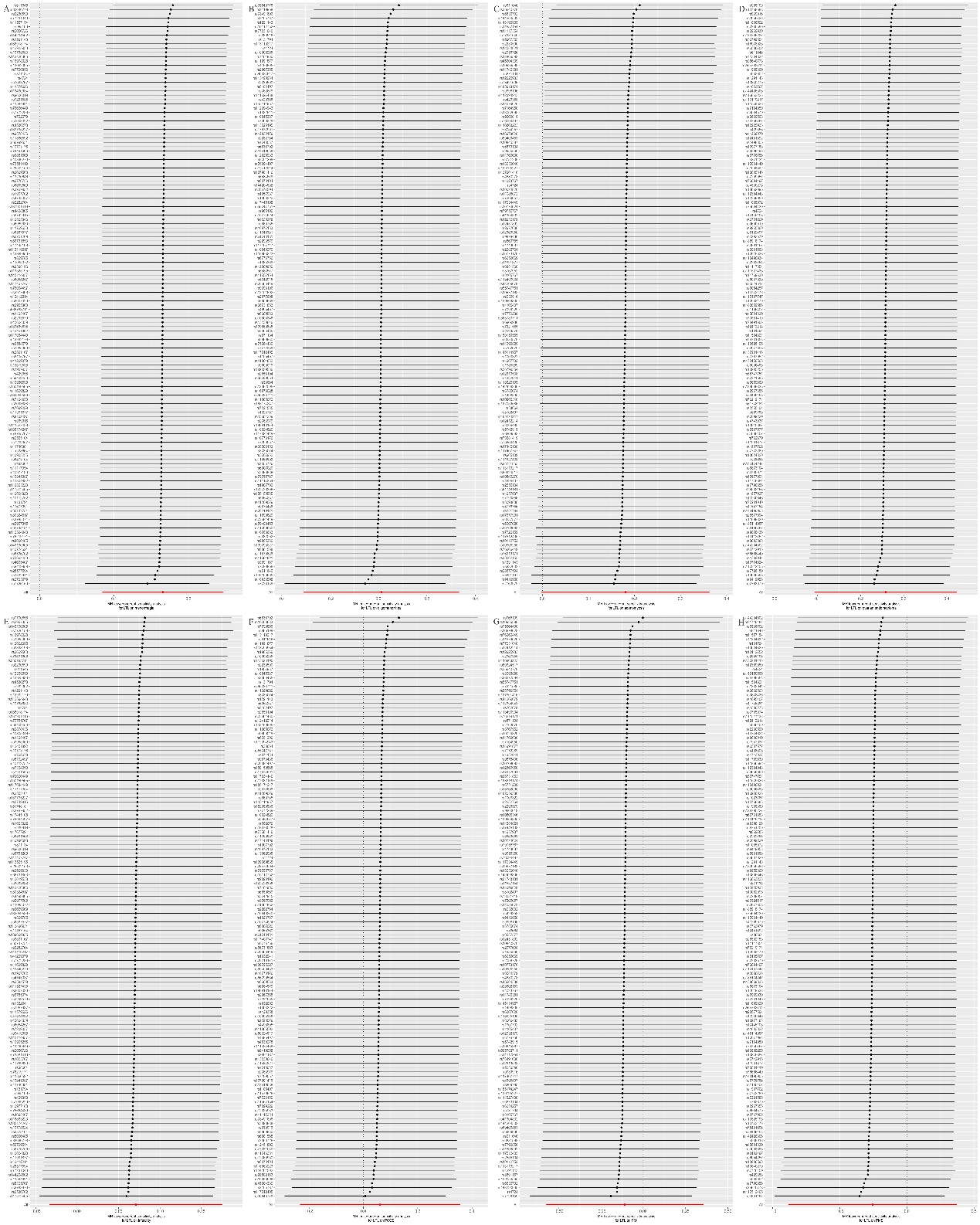

Figure S3. Leave-one-out plots of MR results for the relationship between female reproductive endocrine diseases and LTL. (A)menorrhagia; (B)oligomenorrhea; (C)adenomyosis; (D)ovarian endometrioma; (E)infertility; (F)polycystic ovary syndrome (PCOS); (G)premature ovarian insufficiency (POI); (H)premenstrual syndrome (PMS).
